# A poor outcome in non-occlusive thrombo-embolic limb ischaemia related to the dislocation of mural thrombus from an abdominal aortic aneurysm

**DOI:** 10.1186/s12872-022-02678-7

**Published:** 2022-06-18

**Authors:** Ying-Sheng Li, Ying-Ching Li

**Affiliations:** 1 Division of Thoracic and Cardiovascular Surgery, Department of Surgery, Chang Gung Memorial Hospital at Linkou, Chang Gung University, Taoyuan, Taiwan; 2 Division of NeuroSurgery, Department of Surgery, Chang Gung Memorial Hospital, Linkou, Chang Gung University, Taoyuan, Taiwan

**Keywords:** Acute limb ischaemia, Abdominal aortic aneurysm, Acute thrombosis, Mural thrombosis

## Abstract

**Background:**

Acute thrombosis of an abdominal aortic aneurysm with acute limb ischaemia is an unusual complication and is associated with high mortality. Dislocation of the intrasaccular mural thrombus could be one of the mechanisms. For the most part, acute limb ischaemia presents with absent pulses, compatible with the clinical findings, which include pain, paraesthesia, and paralysis. Herein, we report a rare condition with detectable distal pulses in advanced limb ischaemia due to poor perfusion caused by the dislocation of mural thrombus from an abdominal aortic aneurysm.

**Case presentation:**

A 74-year-old male patient with underlying hypertension and chronic renal disease presented at the emergency room with bilateral lower limb paralysis after falling on his back in the bathroom an hour prior. He reported numbness and weakness of his lower limbs, which was gradually worsening, over the past week. Physical examination showed cyanotic mottling of the lower limbs with paralysis. However, the dorsalis pedis pulse was intact. Computed tomography angiography showed a 7.3 cm abdominal aortic aneurysm containing highly irregular mural thrombus in the early phase, with slow perfusion of the contrast medium in the arteries below the bifurcation during the delayed phase. After traumatic spinal injury was excluded, an emergent endovascular aneurysm repair was performed. Although vital signs were initially stable post-surgery, both lower limbs were still paralysed and did not improve. He then experienced reperfusion injury with metabolic acidosis. There was no urine output despite intravenous hydration. Laboratory data included potassium 7.7 mEq/L, lactate 110 mg/dL, white blood cells 23,700/uL, and myoglobin 46,590 ng/mL. Even under critical medical care and continuous venovenous hemofiltration, his hemodynamic status worsened. He developed hypotension and needed endotracheal intubation because of loss of consciousness and respiratory failure. The patient finally died due to ventricular tachycardia even after several rounds of cardiopulmonary resuscitation with cardioversion.

**Conclusion:**

The unusual clinical presentation of detectable lower limb pulses in advanced limb ischaemia showed that poor blood perfusion related to dislocation of mural thrombus in abdominal aortic aneurysm might mislead clinicians and delay accurate diagnosis and treatment.

## Background

Acute thrombosis of an abdominal aortic aneurysm (AAA) with acute limb ischaemia is an unusual complication which was first reported and successfully treated by Jannetta and Roberts in 1961 [1]. In addition, it is also associated with high morbidity and mortality (45–53%) and poor outcomes, similar to that of ruptured AAAs, according to the literature [2, 3]. For the most part, patients present with symptoms of acute limb ischaemia, including pain, poikilothermia, paraesthesia, absent pulses, skin mottling, and in more severe cases, paralysis [4].

A few mechanisms have been reported that can explain the relationship between acute limb ischaemia and AAA. These include (1) marked iliac atherosclerosis and occlusion; (2) cardioaortic embolization due to cardiac arrhythmias; and (3) shift in the position of the intrasaccular mural thrombus leading to obstruction of blood flow and aneurysm thrombosis [5, 6].

In this report, we present an unusual case of dislocation of massive mural thrombus in an AAA leading to acute limb ischaemia without causing total occlusion of the aortoiliac tract.

## Case presentation

**Fig. 1:**
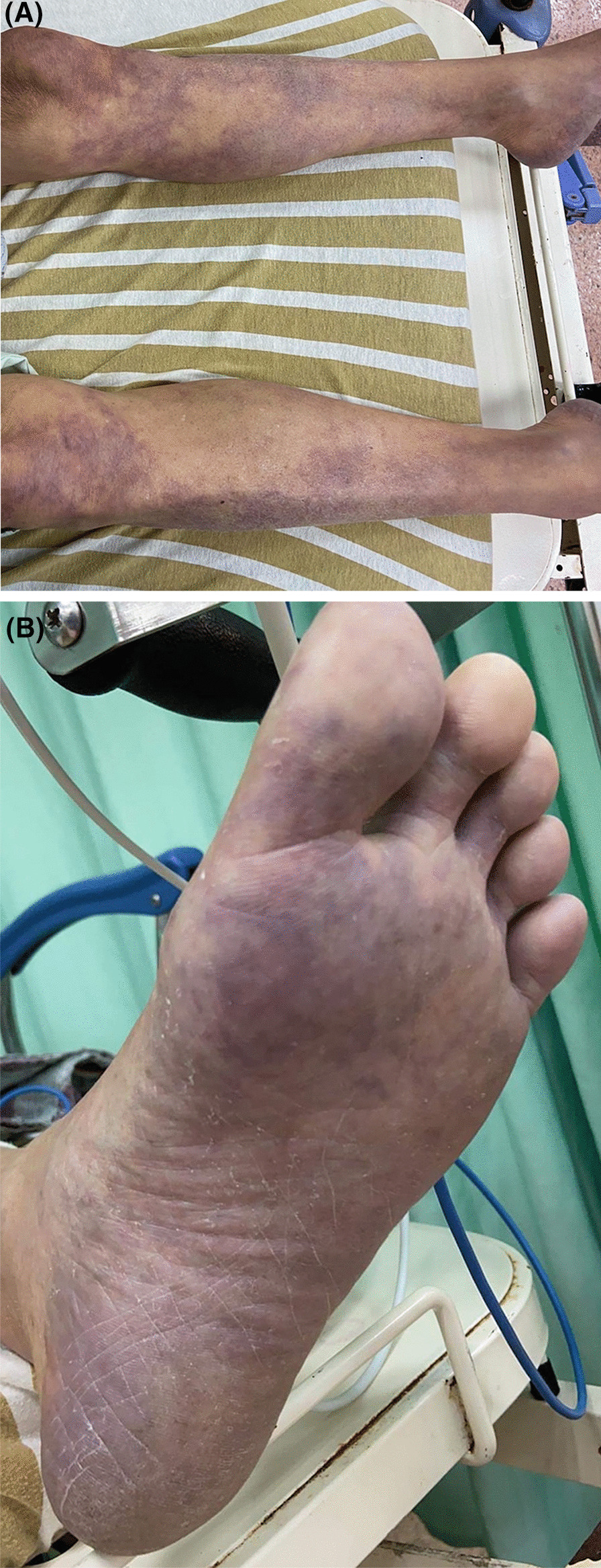
**A** Clinical picture showing bilateral acute lower limb ischaemia with cyanotic mottling of the skin and paralysis. **B** Severe blue toes syndrome was noted in the initial presentation

**Fig. 2:**
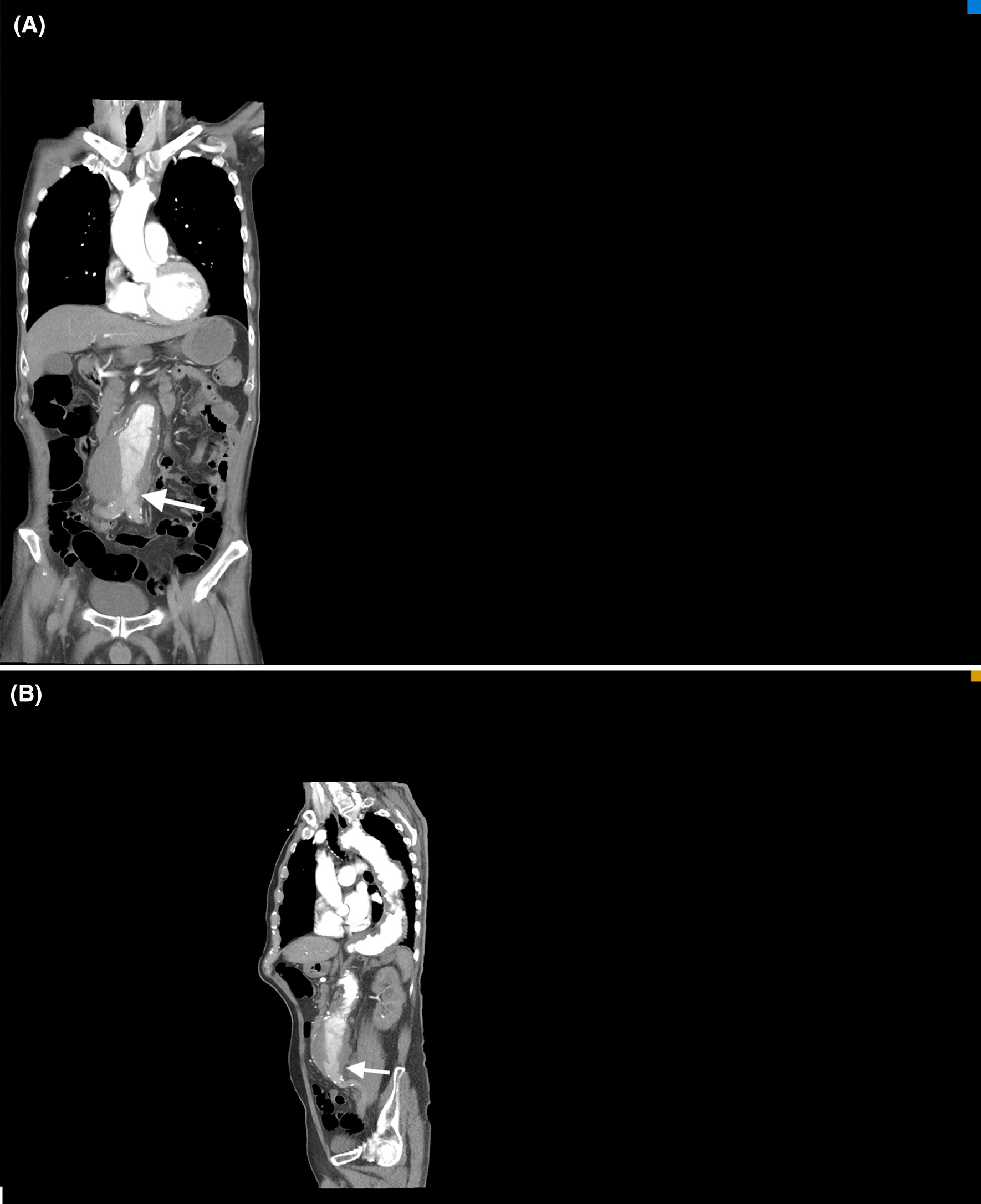
CTA in arterial phase was performed **A** with no contrast medium in the inferior vena cava in coronal view. There was no signal detection below the aortoiliac bifurcation. **B** Sagittal view revealed highly irregular mural thrombus lobulated in the aneurysm with focal flap formation related to dislocation of chronic mural thrombus burden (The both white arrows show a floating flap of thrombus near the aortic bifurcation)

**Fig. 3:**
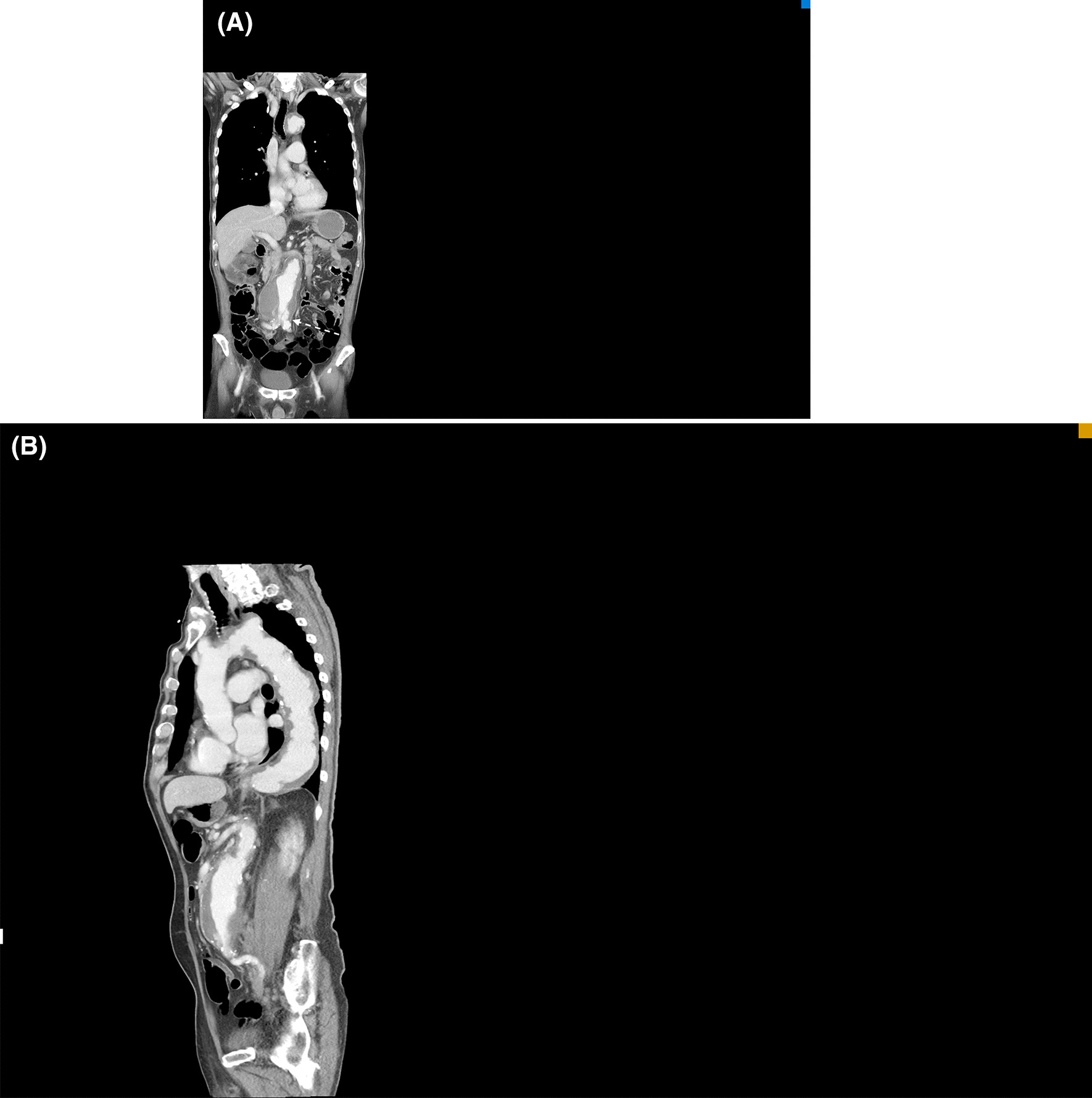
In the delayed phase of CTA, **A** contrast medium was detected in the venous system, shown in coronal view, and there was a signal was apparent in the common femoral arteries. **B** There was patent blood flow in the iliac arteries in which non-occlusive acute limb ischaemia was documented caused by poor inflow (The dotted arrow shows partial occlusion related to a floating flap of thrombus, without embolic occlusion)

**Fig. 4:**
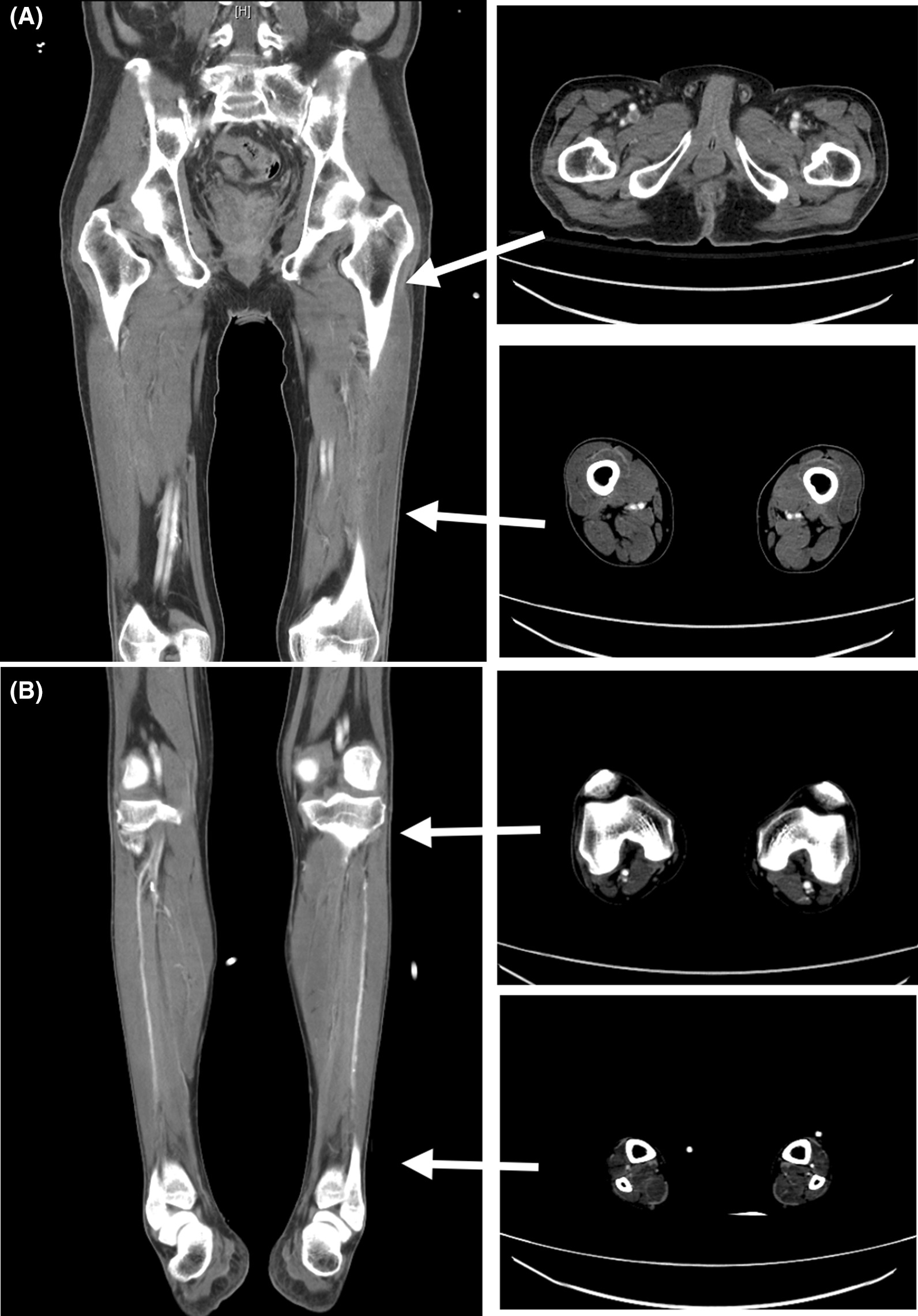
In the lower extremity CTA series, delayed artery flow was detected from the proximal leg to the distal site. **A** No specific embolic lesion was found from the common femoral artery to the distal superficial femoral artery. **B** A similar finding was documented in the distal part of the extremities: patent artery flow without occlusion in the popliteal and below knee arteries

A 74-year-old male patient with underlying hypertension and chronic renal disease presented to the emergency room (ER) with bilateral lower limb paralysis after falling on his back in the bathroom an hour prior. He was a non-smoker, and was not taking his medication regularly. He had no past surgical history. The patient denied any impact to the head or neck, or loss of consciousness. He reported experiencing numbness and weakness of both lower limbs, which was gradually worsening, for more than a week. Physical examination showed cyanotic mottling of the entire skin of the lower limbs with paralysis and loss of sensation below the fifth lumbar dermatome (Fig. 1). In addition, severe blue toe syndrome was suspected due to scattered areas of petechiae and cyanosis of the plantar and dorsal aspects of the feet. However, the pulses of the dorsalis pedis and distal posterior tibial arteries, detected by bedside Doppler ultrasonography, were intact. Computed tomography angiography (CTA) showed a 7.3 cm AAA containing a highly irregular mural thrombus (Fig. 2). Diffuse atherosclerotic changes were present along the entire aortic wall, from the aortic arch to the bifurcation of the abdominal aorta, with multiple aneurysmal outpouchings at the arch. In addition, we detected the contrast medium in the arteries below the bifurcation during the delayed phase of CTA (Fig. 3). In the lower extremities CTA series (Fig. 4), the entire arterial route was patent, without embolic occlusion, from the common femoral artery to the below knee triple vessels. A neurosurgeon was consulted to ascertain whether traumatic spinal injury could have caused this acute-onset paraplegia. However, spinal CT confirmed the absence of lumbar spinal injury. A provisional diagnosis of AAA with a non-occlusive mural thrombus causing acute bilateral lower limb ischaemia was made, and emergent endovascular aneurysm repair (EVAR) was performed for immediate revascularization.

After the EVAR procedure, the patient was conscious and could obey commands, but still could not move his lower limbs. Muscle power was not restored after the procedure. However, pulsation of the bilateral dorsalis pedis and posterior tibial arteries was detected, even though the limbs were still cold, and the skin was pale and cyanotic. Although the patient was hemodynamically stable after surgery and did not require any inotropic support, he received critical care to combat reperfusion injury. A continuous intravenous infusion of sodium bicarbonate was administered to correct metabolic acidosis. With intravenous hydration, using crystalloid fluids administered at the rate of 100 mL/h, urine output of more than 1 mL/kg/min was expected. However, no urine output was recorded after the surgery, even after massive fluid infusion, and the patient developed hyperkalaemia. Acute renal injury was suspected, and continuous venovenous hemofiltration was immediately performed. Lab data obtained at post-operative hour (POH) 6 painted a grim picture with the following results: potassium 7.7 mEq/L, lactate 110 mg/dL, white blood cells 23,700/uL, and myoglobin 46,590 ng/mL. Due to persistent metabolic acidosis and a high lactate level combined with an increase in abdominal circumference, abdominal CT was performed at POH 9 to rule out ischaemic bowel injury. After acute thrombus-related bowel ischaemia was excluded, critical care was continued due to the patient’s worsening medical condition. For a time, the potassium level declined to 5.5 mEq/L at POH 12 with improvement in metabolic acidosis. However, his general condition rapidly worsened during POHs 15–18. The patient developed severe hypotension despite receiving high-dose inotropic support. An endotracheal tube was inserted because of sudden loss of consciousness and respiratory failure. The potassium level also rose rapidly to 9.0 mEq/L resulting in ventricular tachycardia. Unfortunately, the patient died even after several rounds of cardiopulmonary resuscitation with cardioversion.

## Discussion and conclusions

Acute thrombosis of an AAA is a rare and often devastating complication. It is most commonly associated with acute lower limb ischaemia which typically presents with absent femoral pulses [4]. In this patient, an unusual clinical finding was detectable bilateral pulses from the femoral to the distal dorsalis pedis arteries. This was compatible with the CTA findings, even though both lower limbs were completely paralysed and cyanosed. Initially, a neurosurgeon was consulted to ascertain the cause of paraplegia since the patient had presented after falling down at home. This created additional problems for the medical staff in the ER because the unusual presentation was misleading, causing delay in the diagnosis of the underlying medical condition i.e., AAA. After the exclusion of traumatic spinal injury, a vascular surgeon was consulted for pallor and mottling of the skin because Rutherford stage III acute limb ischaemia was suspected. Patients in such a critical medical situation must undergo surgery soon after a definite diagnosis of acute aortic occlusion is made since a delay in treatment may be fatal [3].

Although the patient died even after the emergent EVAR procedure, his CTA was reviewed repeatedly to understand the atypical clinical picture. Out of all the possible mechanisms mentioned in a prior study [5], the main cause of acute limb ischaemia was thought to be a shift in the position of the intrasaccular mural thrombus leading to obstruction or decrease in the blood outflow rate. In addition, there might have been a relationship between the patient's fall, which could have dislocated the thrombus, and the onset of acute limb ischaemia, as reported in some prior case reports [5]. Nevertheless, there was no complete occlusion of the aortoiliac tract, and no obvious thromboembolic lesion was found on CTA. In fact, the dislocation of the massive thrombus led to the narrowing of the aortoiliac tract. This resulted in the reduction of blood flow, which caused ischaemia due to poor perfusion. The mechanism of slow flow contributes to peripheral arterial disease manifestations as well as the slow flow phenomenon in the era of widespread coronary system. The putative mechanism is shower embolism of tiny clots or particles to small peripheral arterial system. In addition, concomitant heart failure, which is commonly associated with abdominal aortic aneurysm and severe peripheral vascular disease has a major role in aggravating the slow flow in lower extremities. Therefore, the clinical presentation was atypical because detectable bilateral dorsalis pedis pulses, compatible with CTA findings, were present, despite bilateral lower limb paralysis and cyanosis. In the arterial phase of CTA (Fig. 2), contrast medium was not detected in the blood vessels below the aortic bifurcation. Initially, aortoiliac occlusion was suspected; however, the iliac and femoral arteries were patent without occlusion as seen during the delayed phase of CTA (Fig. 3). Finally, a diagnosis of bilateral acute lower limb ischaemia related to poor blood perfusion caused by the dislocation of the massive non-occlusive mural thrombus in the AAA was made.

Even after appropriate treatment, acute thrombosis of an AAA is associated with a high mortality rate [3]. In this case, a rare finding of detectable pulsation in Rutherford stage III acute limb ischaemia showed that poor blood perfusion was related to dislocation of the mural thrombus that did not cause complete occlusion. In addition, we concluded that the unusual clinical presentation of acute limb ischaemia might mislead clinicians and cause delays in diagnosis and treatment. Clinicians should keep in mind that poor perfusion can cause ischaemia even without complete occlusion of the aortoiliac tract.

## Data Availability

The raw data is not publicly available due to the medical record.

## Abbreviations

AAA: Abdominal aortic aneurysm
ER: Emergency room
CTA: Computed tomography angiography
EVAR: Endovascular aneurysm repair
POH: Post-operative hour
